# Innate immunity in Alzheimer’s disease: the relevance of animal models?

**DOI:** 10.1007/s00702-017-1729-4

**Published:** 2017-05-17

**Authors:** Diana K. Franco Bocanegra, James A. R. Nicoll, Delphine Boche

**Affiliations:** 1 Clinical Neurosciences, Clinical and Experimental Sciences Academic Unit, Faculty of Medicine, University of Southampton, Southampton General Hospital, Mailpoint 806, Southampton, SO16 6YD UK; 2 Department of Cellular Pathology, University Hospital Southampton NHS Foundation Trust, Southampton, Southampton, SO16 6YD UK

**Keywords:** Microglia, Human brain, Animal model, Alzheimer’s disease

## Abstract

The mouse is one of the organisms most widely used as an animal model in biomedical research, due to the particular ease with which it can be handled and reproduced in laboratory. As a member of the mammalian class, mice share with humans many features regarding metabolic pathways, cell morphology and anatomy. However, important biological differences between mice and humans exist and must be taken into consideration when interpreting research results, to properly translate evidence from experimental studies into information that can be useful for human disease prevention and/or treatment. With respect to Alzheimer’s disease (AD), much of the experimental information currently known about this disease has been gathered from studies using mainly mice as models. Therefore, it is notably important to fully characterise the differences between mice and humans regarding important aspects of the disease. It is now widely known that inflammation plays an important role in the development of AD, a role that is not only a response to the surrounding pathological environment, but rather seems to be strongly implicated in the aetiology of the disease as indicated by the genetic studies. This review highlights relevant differences in inflammation and in microglia, the innate immune cell of the brain, between mice and humans regarding genetics and morphology in normal ageing, and the relationship of microglia with AD-like pathology, the inflammatory profile, and cognition. We conclude that some noteworthy differences exist between mice and humans regarding microglial characteristics, in distribution, gene expression, and states of activation. This may have repercussions in the way that transgenic mice respond to, and influence, the AD-like pathology. However, despite these differences, human and mouse microglia also show similarities in morphology and behaviour, such that the mouse is a suitable model for studying the role of microglia, as long as these differences are taken into consideration when delineating new strategies to approach the study of neurodegenerative diseases.

## Introduction

Since the establishment of the amyloid cascade hypothesis to explain Alzheimer’s disease (AD) by John Hardy in 1992 (Hardy and Higgins 1992), more than 110 mouse models of AD have been developed (Alzforum database), mainly based on point mutations identified in familial/early onset AD. This high number of models demonstrates the challenge associated with the modelling of a complex human disease in rodents. The majority of these models reproduce amyloid-β (Aβ) protein deposition as the animal is ageing, with the presence of some other components of AD pathology such as microglial activation, neuronal loss (although not to the same extent as in humans), and memory impairment (LaFerla and Green 2012). One of the difficulties of modelling AD pathogenesis is the ability to reproduce in association with Aβ accumulation, a robust accumulation of phosphorylated tau protein that takes years or decades to accumulate in humans, in the limited lifetime of a laboratory rodent.

Microglia, as the tissue-resident macrophages in the brain, belong to the mononuclear phagocyte cell population (Boche et al. 2013). The presence of activated microglia clustering around amyloid plaques is a constant observation in both animal models and the human brain. However, their role in the pathogenesis is still debated as whether microglia are responding to or contributing to the neurodegenerative process, or both. Experimental studies have shown that microglia derive from the yolk sac (Ginhoux et al. 2010) and differ from the other macrophage populations in having a distinct homeostatic signature leading to the expression of microglial-specific genes (Butovsky et al. 2014). During brain development, microglia are critically involved in pruning overabundant synapses leading to the organisation and maturation of neuronal networks (Bilbo and Schwarz 2012; Schafer et al. 2012; Squarzoni et al. 2014). In the adult mouse brain, they remain dynamic cells surveying the microenvironment (Nimmerjahn et al. 2005) and participating in synaptic plasticity (Wu et al. 2015). Interestingly, recent studies reported sexual dimorphism in microglial behaviour in the developing neonatal brain (Lenz et al. 2013) and in pathological conditions (Mirza et al. 2015), potentially explaining the gender-associated susceptibility observed in some neurodevelopmental and neurodegenerative disorders (Hanamsagar and Bilbo 2016), including psychiatric conditions (Werling et al. 2016). Pathological experimental conditions have demonstrated that macrophages, and thus microglia, are highly plastic cells, expressing a range of inflammatory molecules, ranging from the pro-inflammatory/potentially deleterious to the anti-inflammatory/potentially beneficial spectrum based on the stimulus (Boche et al. 2013). As a result, microglial activation in neurodegenerative diseases has traditionally been considered simply as a reaction to the ongoing neurodegeneration. However, with recent evidence, this view is changing. For example, clinical studies have shown that patients affected by a systemic infection decline more rapidly (Holmes et al. 2003, 2009), and it is now recognised that systemic inflammation is an important component of human neurodegenerative diseases (Holmes 2013). This is essential information to take into account when studying disease in animals kept in a clean specific-pathogen-free atmosphere, quite different from our daily “dirty” environment. The consequence of repetitive systemic infections on microglia is that they become “primed” and thus more susceptible to activation (Perry et al. 2010; Perry and Holmes 2014), acquiring an innate memory (Netea et al. 2015).

As mentioned above, many animal models of AD are based on mutations associated with rare familial forms of the disease (e.g., APP, PS1, and PS2 mutations) and few encompass the main risk factors of the more numerous late-onset/sporadic AD population.

Ageing and polymorphism of the apolipoprotein E (APOE) gene are the main risk factors for AD (McDowell 2001; Spinney 2014). Genome-wide association studies have also highlighted several inflammation-related genes as risk factors for late-onset AD, particularly in relation to innate immunity. This indicates that a component of microglial activity is likely to be involved in the onset and/or the progression of the pathological events (Harold et al. 2009; Lambert et al. 2009; Jones et al. 2010a; Guerreiro et al. 2012; Jonsson et al. 2012), as well as potential peripheral immune components, further highlighting the limitations of current animal models.

In this review, we highlight some of the differences observed between mouse and human microglia in the context of ageing and AD.

## Genetic studies of microglia

In contrast to rare familial early onset AD, common sporadic late-onset AD is not caused by genetic abnormalities. Despite this difference, many studies have shown that genetic factors are important in the development of sporadic AD, as many polymorphisms have been found to modify the risk of the disease (Table 1). Therefore, as AD is a complex disease in which genes are known to play an important role, an ideal model organism for AD would be one whose genome closely resembles the human in the expression of key genes.

**Table 1:** Genetic factors in Alzheimer’s disease

Early onset/familial Alzheimer’s disease			Late-onset/sporadic Alzheimer’s disease	
Gene	Mutation	Pathology	Gene	Function of encoded protein
APP	KM670/671NL (Swedish)	Increased total Aβ including production and secretion of Aβ42 and Aβ40 (Mullan et al. 1992)	APOE	Catabolism of triglyceride-rich lipoprotein constituents. A role in Aβ aggregation and clearance (Corder et al. 1993)
	D694N (Iowa)	Extensive cerebral amyloid angiopathy; Widespread neurofibrillary tangles; Increased fibrillogenesis of the Aβ peptide (Grabowski et al. 2001)	BIN1	Adaptor protein potentially involved in synaptic vesicle endocytosis (Hu et al. 2011)
	V717I (London)	Mild amyloid angiopathy; numerous plaques and tangles. Increased Aβ42/Aβ40 ratio; increased Aβ42 (Goate et al. 1991)	CLU	Secreted chaperone involved in apoptosis and complement regulation (Harold et al. 2009)
PS1	M146V	Increased Aβ42/Aβ40 ratio; increased Aβ42 (Riudavets et al. 2013)	ABCA7	Member of the superfamily of ATP-binding cassette (ABC) transporters, which transport molecules across membranes. Potential role in lipid homeostasis in immune cells (Hollingworth et al. 2011)
	M146L (A>C)	Increased Aβ42/Aβ40 ratio; increased Aβ42 (Shioi et al. 2007)	CR1	Mediates cellular binding to particles and immune complexes via activated complement (Corneveaux et al. 2010)
	L286 V	Increased Aβ42/Aβ total ratio (Frommelt et al. 1991)	PICALM	Recruits clathrin and adaptor protein complex 2 (AP2) to cell membranes at sites of coated-pit formation and clathrin-vesicle assembly (Harold et al. 2009)
	L166P	Numerous Aβ-positive neuritic plaques throughout the cerebral cortex; Increased Aβ42/Aβ ratio (Moehlmann et al. 2002)	MS4A6A	Likely involved in activation of T cells (Hollingworth et al. 2011)
PS2	N141I	Extensive amyloid plaques; Extensive neurofibrillary tangles (typically a Braak score of V or VI); α-synuclein inclusions, especially in the amygdala; Hippocampal sclerosis. Increased Aβ42/Aβ40 ratio; increased Aβ42 (Jayadev et al. 2010)	CD33	Transmembrane receptor expressed on myeloid cells (Hollingworth et al. 2011)
	M239I	Moderate cortical atrophy; Numerous neurofibrillary tangles; Numerous senile plaques, especially in the amygdala. Increased Aβ42/Aβ40 ratio; increased Aβ42 (Finckh et al. 2000)	CD2AP	Scaffolding molecule that regulates the actin cytoskeleton. Interacts with filamentous actin and various cell membrane proteins through multiple actin-binding sites Implicated in actin remodelling and membrane trafficking that occurs during receptor endocytosis and cytokinesis, and thus involved in cell motility (Hollingworth et al. 2011)
	M239 V	Numerous neurofibrillary tangles (Braak stage VI) in addition of plaques; Extracellular “ghost” neurofibrillary tangles (Marcon et al. 2004)	TREM2	Gateway influencing balance between phagocytic and pro-inflammatory microglial activity (Jonsson et al. 2012)
			HLA-DR5, DBR1	Immunocompetence, involved in antigen presentation (Hamza et al. 2010)

Some of the most important genetic factors associated with AD. In familial AD, mutations are causal, while in sporadic AD, polymorphisms in the listed genes among others have been identified as risk factors

The human and mouse genomes have significantly diverged from a common ancestor (Hedges et al. 2006). There is evidence that only around half of the human genomic DNA can be aligned to the mouse genomic DNA (Waterston et al. 2002), and expression profiles of both species vary considerably. Although there is a relatively high degree of conservation, many mouse genes show different expression profiles compared to their human orthologues, and the level of divergence varies depending on tissue type (Yue et al. 2014). It was established that these differences in gene expression could be explained mainly by the inter-species divergence in sequence of cis-regulatory elements (Yue et al. 2014). Regulation of gene expression is an important aspect that determines cell phenotype and behaviour. When working with a model organism, tissue-specific differences in patterns of gene transcription may have an impact on how the organism responds to experimental procedures and, therefore, should be considered when designing experiments and interpreting research results.

To assess the similarities and differences in gene expression patterns between mice and humans, Miller et al. (2010) evaluated the coexpression of genes in the brain of both species and established clearly delineated modules (groups of highly coexpressed genes) that closely depict defined biological processes (e.g., cytoskeletal organisation and mitochondrial function). They found modules of interacting genes highly conserved across species, meaning that both mice and humans share similar patterns of gene expression. Interestingly, their results also pointed out that modules corresponding to proteins expressed by neurons showed a higher level of conservation between species than modules corresponding to astrocytes or microglia, which were mainly composed of genes that participate in the immune response. Hence, inflammatory genes are the ones exhibiting the most inter-species divergence (Miller et al. 2010).

These findings are of particular importance, since among the AD-associated polymorphisms that have been identified in case–control association studies or in GWAS, several occur in genes that are related to the immune response and inflammation (Harold et al. 2009; Lambert et al. 2010; Jones et al. 2010b; Guerreiro et al. 2012; Jonsson et al. 2012). Inflammation is an important factor strongly linked to the neurodegenerative process; and thus, it is worth noting that the expression rate of inflammation-related genes may be determinant in developing the pathology and symptoms that are characteristic of the disease and it may be a key feature in the effectiveness and accuracy of a disease model. Therefore, the difficulty associated with the reproduction of human AD, e.g., symptoms and pathology, in animal models might be explained partly by the divergence in the expression of glial genes. Surprisingly, the study by Miller also found significant differences in brain expression in other AD-related proteins, such as glycogen synthase kinase (GSK)-3β, tau, and presenilin-1. The evidence that mice and humans show divergent gene expression patterns precisely in genes related with AD and other dementias is remarkable as it highlights the importance of the pathways that these genes are implicated in, and sheds some light over the possible reasons for which the mouse brain has not been able to fully replicate all hallmarks of AD pathology.

Considering all of the above, it has been suggested that the microglial transcriptome might account for the main inter-species differences in the brain transcriptome (Miller et al. 2010). Therefore, studying the specific microglial gene expression signature could provide insight on how the central immune response differs from the macrophage response in other body systems, and on the manner, the mouse and human respond differently to neurotoxic insults such as protein aggregation. Recent evidence supports the idea that microglia are a defined cell type morphologically and functionally different from other glial cells and monocyte-derived cells. Butovsky et al. (2014) compared the mouse microglial gene expression with that of other major brain cell populations, e.g., astrocytes, oligodendrocytes, and neurons, and they identified 106 genes specifically expressed in microglia the expression of which is driven by the cytokine transforming growth factor (TGF)-β. When comparing their data with human microglia, they observed that several human microglial genes were not expressed in mice. Interestingly, the most highly expressed gene in murine microglia was *Fcrls*, for which there is no orthologue in the human genome (Butovsky et al. 2014). The Fc receptor-like S, also known as the immunoglobulin scavenger receptor or macrophage scavenger receptor 2, is associated with the M2 activation profile of macrophages during brain development (Rae et al. 2007) and in a mouse model of brain tumour (Biswas et al. 2006). This suggests that Fcrls may have a trophic and phagocytic function.

It is important to note that the regulation of gene expression is not constant in the organism, but, on the contrary, undergoes a variety of changes throughout the lifetime. Several authors have documented that in humans, as well as in other species, specific changes are associated with ageing. These changes include differences in RNA splicing and processing, and in chromatin assembly and disassembly, among others (Harries et al. 2011). Considering all of the above and since epidemiological data show that ageing is the most important risk factor for sporadic AD, the comparison of the gene expression profile between young and aged organisms can provide insight as to which genes and metabolic pathways are associated with ageing both in healthy and pathological conditions.

Studies on the mouse brain transcriptome have revealed that this species displays significant changes in gene expression during ageing. Lee et al. (2000) have assessed the expression of 6347 genes in the neocortex and cerebellum of young and aged mice. They detected an increased expression of several genes during ageing, with more than 20% of these genes related to the inflammatory response. The observations of altered regulation of genes involved in neuroinflammation during ageing have been since confirmed by a number of recent publications. Indeed, a study in both mice and humans showed that ageing is normally accompanied by an increased expression of cytokines such as interleukin (IL)-1β, IL6, tumour necrosis factor (TNF)-α and TGFβ, chemokines, complement proteins, and toll-like receptors (TLRs) suggesting a similar age-related inflammatory landscape in the brains of both species (Lopez-Gonzalez et al. 2015). Another study evaluated gene expression of microglia isolated from young and aged mice, and found an ageing-related increased expression of genes from the TNF ligand superfamily (Orre et al. 2014). Interestingly, a decreased expression of several proteins associated with chemotactic response, such as integrins, chemokines of the CXC family and their receptors, adhesion molecule receptors, and the heparin-binding EGF like growth factor was also observed. This indicates that with ageing, the inflammatory state is exacerbated, but at the same time associated with an impaired response to injury.

It is worth noting that, in both species, there is a marked increase in the expression of pro-inflammatory genes during normal ageing. Interestingly, in humans, the changes in inflammatory genes were significantly higher when comparing young with aged individuals than when comparing non-demented aged subjects with AD subjects (Cribbs et al. 2012). These findings coincide with those of Tollervey’s group who evaluated changes in transcript levels and alternative splicing in the brains of healthy subjects of different ages and subjects affected with AD. They observed that the gene expression differences were more pronounced between young and aged people than between cognitively normal ageing and dementia (Tollervey et al. 2011).

Although there are similarities in the way that gene expression changes during normal ageing in both species, there are also some marked differences. Evaluating expression profiles of transgenic APPSwePSEN1dE9 mice showed that in general, the immune response seems to be broader in mice than in humans, as more inflammatory genes displayed increased expression in pathological conditions, and inflammatory changes coincided largely with amyloid deposition in the form of plaques. In humans, these changes have been associated preferentially with the presence of oligomeric Aβ rather than amyloid plaques (Lopez-Gonzalez et al. 2015).

From these studies, it appears that mice are organisms with a genetic background that broadly resembles that of humans in the fact that many genes and proteins are conserved between both species. However, the mouse gene expression patterns show important differences, particularly in inflammation-related genes that should be taken in consideration when interpreting observations from mouse models and their implications in the context of neurodegeneration in human diseases.

## Morphology, location, and role of microglia

The mouse and human central nervous systems (CNS) are different not only in their molecular aspects but also in their anatomical structure. One important difference is the distribution of grey versus white matter in the brain. In mammalian brain, white matter volume increases faster than that of the grey matter as the total brain size augments (Zhang and Sejnowski 2000). As a result, the human brain has a higher white/grey matter ratio compared to the mouse brain. In humans, the white matter represents 45–50% of total brain volume, while in the mouse, it accounts only for around 10% (Gur et al. 1999; Zhang and Sejnowski 2000).

Regarding the distribution of microglial cells, the above-mentioned facts are important for the density of microglia which varies between grey and white matters, and also between species. In mice, microglial density is higher in grey matter (Lawson et al. 1990; Hart et al. 2012); while in humans, microglia are more numerous in white matter (Hart et al. 2012; Mittelbronn et al. 2001). Therefore, due to the abundance of white matter in the human brain, this implies a relatively higher overall microglial cell population in humans than in mice. Microglial density differs not only between grey and white matter but also between different brain regions, ranging from 0.3% in the grey matter of the cerebellum to 16.9% in white matter of medulla oblongata in humans (Mittelbronn et al. 2001). The relatively high microglial density in the human CNS makes it more likely to be affected by an impaired or deleterious phenotype developed by microglia, an effect that might not be so marked in the mouse brain.

Experimental studies have shown that ageing modifies the microglial distribution within the brain, from a homogeneous distribution pattern to a heterogeneous one. With ageing, microglial coverage, defined as the volume of tissue under the surveillance of an individual microglial cell—i.e., the volume and its branches are able to reach, is reduced by more than 50% (Baron et al. 2014). This marked change in microglial morphology likely affects the surveillance coverage, suggesting an altered microglial sensing property associated with ageing in human (Davies et al. 2016). Another study using in vivo two-photon microscopy revealed, with ageing, a ~14% increase in microglial number and that microglia were no longer homogeneously distributed throughout the brain tissue (Hefendehl et al. 2014), consistent with the study by Baron and colleagues. Interestingly, a recent study exploring the density of microglial cells in white and grey matter found no changes associated with ageing in either mouse or human brains (Askew et al. 2017). Overall, these studies on ageing in the mouse brain provide a consensus in the changes in microglial distribution and morphology, but also a discrepancy in the findings related to the cell density.

Several ageing-related changes have been reported regarding microglial phenotype and functional properties. Observations from the mouse brain show a thickening and a deramification of microglial processes with hypertrophy of the cell body. These phenotypic changes coincide with an increased expression of CD68, CD11b, CD11c, and FcγRI (CD64). Interestingly, the expression of these microglial markers and the changes in morphology were reported to be more extensive in white matter than in grey matter (Hart et al. 2012).

**Fig. 1:**
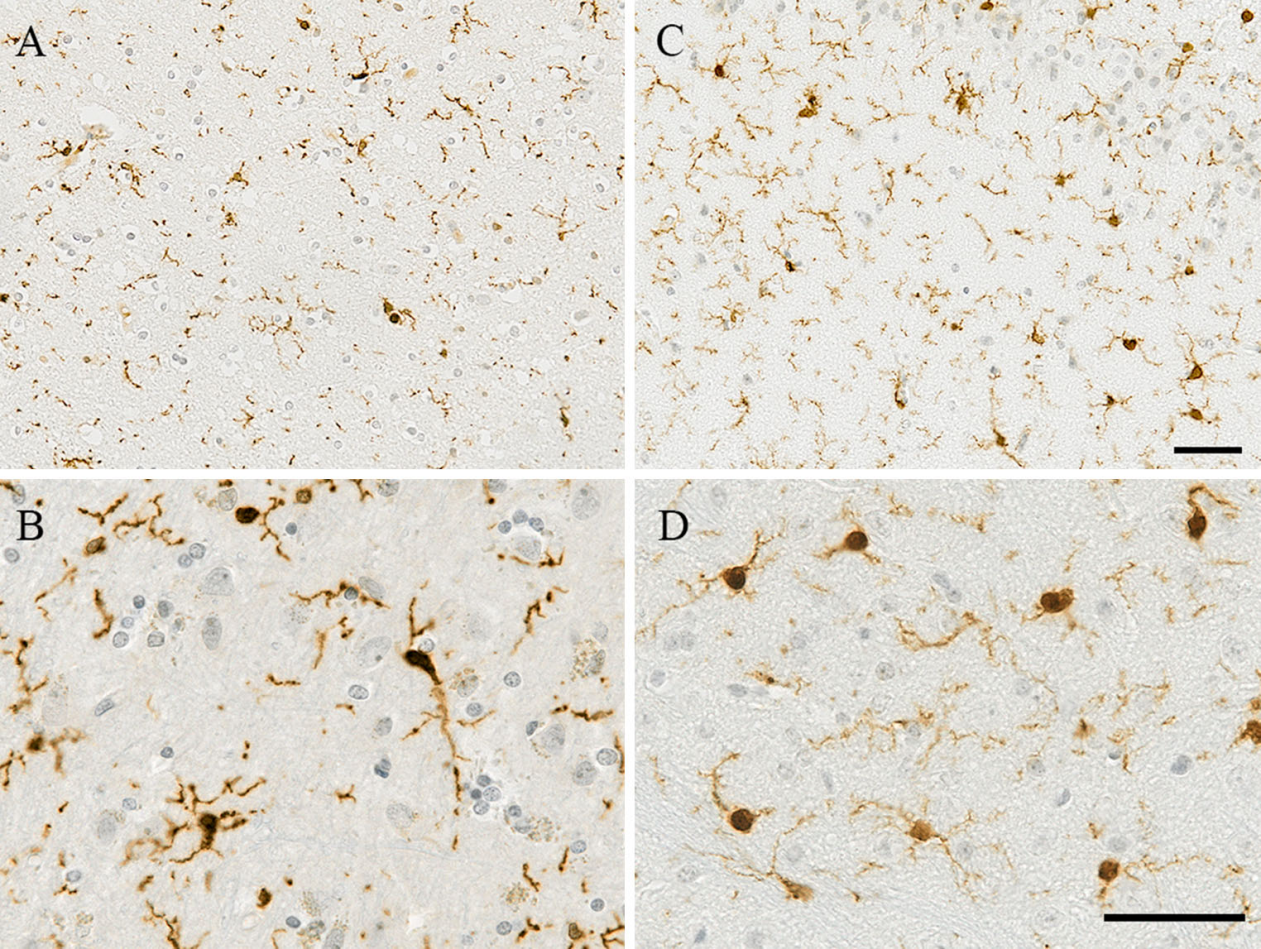
Images of human and mouse microglia immunostained with Iba1. **a**–**c** Both species exhibit ramified microglia with several processes. At higher magnification, **b** microglia in the inferior parietal lobe of a human 70-year-old brain show thicker and shorter processes, maybe representative of reactive/primed microglia as described in the literature (formalin fixed paraffin embedded section of 4 μm thickness, post-mortem delay 31 h), compared to **d** the mouse cortical microglia that maintain ramified morphology with finer processes at 52 weeks old (paraformaldehyde 4% fixed paraffin embedded section of 10 μm thickness). Haematoxylin and eosin counterstain. *Scale bar* 50 μm

Studies in mice have identified four morphological phenotypes of microglia (Torres-Platas et al. 2014), supposedly representing different degrees of activation and defined as: ramified, primed, reactive, and amoeboid. The ramified morphology shows a small cell body and several long, thin, ramified processes, and has been referred to as “resting” microglia. Primed microglia exhibit an increased cell body size with unchanged processes and ramifications. Reactive microglia display a more rounded/amoeboid body with a reduced number of processes, but ramifications are still visible (Fig. 1). Finally, amoeboid microglia are completely devoid of ramifications characterised by a spherical body with one or two thickened processes or no processes (Torres-Platas et al. 2014) (Table 2). Curiously, morphometric analysis of microglia using time-lapse imaging in rodents has revealed that in the transition from ramified to the reactive or amoeboid morphology, the ramified processes do not thicken, but rather are reabsorbed into the cell body and replaced by new and enlarged processes with enhanced motility (Stence et al. 2001). At the molecular level, independently of the phenotype of activation, microglia are characterised mainly by the constitutive expression of Iba1, CD68, and MHC-II, which are the most commonly used markers to immunolabel these cells (Mittelbronn et al. 2001).

**Table 2:** Microglial morphologies and their distribution in human and mouse brain (from Torres-Platas et al. 2014)

Morphology	Description	Human (%)	Mouse (%)
Ramified	Small cell body and thin, highly ramified processes	16	90
Primed	Wider cell body but processes are still similarly ramified	34	0
Reactive	Wider and rounder cell body, less ramification	32	6
Amoeboid	Only one or two unramified processes, or no processes at all	18	4

In the past, ramified microglia have been considered to be in a resting, inactive state. However, it is now acknowledged from in vivo two-photon imaging studies in mice that the ramified morphology is actually associated with high motility. Resting microglia show constant motility, which may serve a surveillance function, clearing cellular debris, controlling the brain microenvironment, thereby remodelling the extracellular matrix, and, at the same time, facilitating a timely reaction in the context of a cerebral injury/insult (Nimmerjahn et al. 2005). Recent studies in mice have demonstrated that the motility of microglia is an essential component of neuroplasticity during brain development (Bilbo and Schwarz 2012; Wu et al. 2015) and in the adult (Hanisch and Kettenmann 2007). Neuroplasticity is the ability of the brain to compensate and adjust its activity in response to changes in the environment by an alteration in the neurons or glial cells via cell division/apoptosis and/or synaptic/neurite alterations remodelling the neuronal network. Constant surveillance of the microenvironment by microglial processes (Hanisch and Kettenmann 2007) and their attraction to active rather than non-stimulated synapses implies that microglia might monitor the functional state of synapses leading to plastic changes in healthy adult brain (Tremblay et al. 2010). Microglial motility relies mainly on cytoskeletal changes based on motile bundles of actin filaments (Lambrechts et al. 2004), a cytoskeletal protein abundantly present in microglia as seen in the rat brain (Capani et al. 2001). One of the microglial proteins also involved in motility Iba1, an actin cross-linking protein crucial for actin bundling and microglial membrane ruffling (Ohsawa et al. 2000), and commonly used as an immunohistochemical marker for microglia.

Findings from biopsies and post-mortem studies confirmed that human microglia also display the same four morphologies described in mice. Particularly, the morphology of ramified microglia is extremely alike between both species (Fig. 1). This similarity in morphology suggests highly conserved functions. Nevertheless, it is interesting to note that the proportion in which these different morphologies are observed in the brain differs between mice and humans. In mice, microglia are mostly ramified cells (>90% of microglial cells) (Torres-Platas et al. 2014), and the reactive and amoeboid morphologies are very scarce (Hart et al. 2012). On the contrary in humans, the most frequent morphology observed is that of primed microglia (34%) (Torres-Platas et al. 2014). The other morphological phenotypes of microglia are also detected in humans in considerable proportions, with 16% ramified cells, 32% reactive cells, and 18% amoeboid cells (Torres-Platas et al. 2014) (Table 2). These observations imply a higher “state of alert”, or a more intense level of activation in human microglia, which, in pathological conditions, could be translated to a higher susceptibility to develop pro-inflammatory profiles contributing to neurodegeneration. The concept of priming is associated with a prolonged and amplified response to immune challenge (Perry and Holmes 2014), potentially leading to a microglial memory. These morphological differences might be partly explained by the environment in which mice and humans live, i.e., “specific-pathogen-free” for the mouse *versus* “dirty” environment for the human, but also by the difference in lifespan.

Interestingly, it was observed in the human AD brain that, apart from the resting and activated morphologies mentioned above, microglia were also exhibiting another abnormal morphology defined as “dystrophic” or “senescent”, characterised by the fragmentation of the microglial processes referred to as “beading” (Streit et al. 2009).

In AD, it is commonly assumed that microglia become activated as the result of amyloid accumulation in the brain, and that this activation is detrimental. Surprisingly, Streit and colleagues noticed that the occurrence of dystrophic microglia preceded the presence of AD pathology and that, once the pathology was present, these cells were mainly associated with tau rather than with amyloid. They showed that Aβ deposits that lack the presence of tau, colocalised with ramified non-activated microglia, implying that microglia might not respond to amyloid accumulation as widely supposed, but to tau (Streit et al. 2009).

Based on these findings, they proposed a novel hypothesis regarding the role of microglia in AD pathology. This was that, rather than microglial activation being harmful, it is an ageing-related microglial dysfunction with the subsequent loss of a microglial neuroprotective effect that actually contributes to neurodegeneration leading to the onset of the disease. This hypothesis poses new challenges and highlights the need to further characterise microglial function in normal and pathological conditions.

## Microglia and AD pathology

As described in the previous section, microglia can adopt different morphologies in normal and pathological conditions, referred to as different states of activation, independently of the species. The relationship between these diverse activated states and the distinctive pathological features of AD—i.e., amyloid plaques and tau accumulation as neurofibrillary tangles (NFTs), dystrophic neurites, and neuropil threads—still remains unclear. For a long time, microglial activation was considered as a physiological response to the protein accumulation and neurodegeneration in AD; but this viewpoint has been challenged in the light of new evidence from genetic studies. Indeed, these novel findings associating polymorphisms in inflammation-related genes with the development of the disease indicate that microglial dysfunction is a factor contributing to the onset of disease in a causal way and not only a response to the neuropathological changes. The lack of understanding of the complex interactions between microglia and AD pathology complicates the replication of the whole disease spectrum in small laboratory animals.

Although there are significant differences between mice and humans, microglia of both species seem to have several points in common regarding morphology and function, thus supporting the suitability of the mouse as a model organism, as long as the limitations of the models are well understood and taken into consideration when interpreting the results.

However, an important difference to highlight regarding AD and ageing is that the mouse brain is not capable of naturally forming amyloid plaques. Consequently, to explore AD-like pathology in mice, transgenic animals that express human point mutations associated with early onset AD (e.g., APP and presenilins) have been developed based on the amyloid cascade hypothesis (Karran et al. 2011). To date, no animal model of AD replicates all of the classical hallmarks of the disease in the same brain in a reliable manner, and this has hampered the development of treatments for late-onset AD (Karran et al. 2011).

Most of the transgenic models of AD are made by combining in the same organism, human transgenes carrying the Swedish double mutation (KM670/671NL) for *APP* and/or different mutations of the *PSEN1* gene. The most common used models are listed and described on Table 3. For example, the APPPS1 mouse strain, a frequently used model of AD, carries the human *APP* gene containing the Swedish mutation and the human *PSEN1* gene bearing the L166P mutation. As mentioned above, each of these mutations is known to cause a familial form of AD, and thus, as a result of the multiple combined mutations, APPPS1 mice show age-related amyloid plaque deposition (Holcomb et al. 1998) and cognitive impairment (Webster et al. 2014). Although they do not develop fibrillary tau pathology, some phosphorylated tau-positive neuritic processes have been observed near amyloid deposits (Radde et al. 2006).

**Table 3:** Main mouse models of Alzheimer’s disease and their pathological features

	Model	AD pathology	Microglia/gliosis	Cognitive impairment
Aβ	APPPS1 (Radde et al. 2006)	Aβ deposition starts at 6 weeks in the cortex and at 3–4 months in the hippocampus Phosphorylated tau-positive neuritic processes around plaques are observed, but no NFTs Modest neuronal loss in the granule cell layer of the dentate gyrus, but overall no neurodegeneration	Activated microglia observed around Aβ deposits at 6 weeks, as well as increased astrogliosis	Cognitive deficits in spatial learning and memory in the Morris water maze at 7 months
	Tg2576 (Hsiao et al. 1996)	Numerous Aβ plaques present at 11–13 months NFTs are absent and there is no neuronal loss	Increased microglial density in plaque-forming areas of the brain including the hippocampus, frontal cortex, entorhinal cortex, and occipital cortex in 10–16-month-old hemizygotes	Reports of impaired spatial learning, working, memory and contextual fear conditioning at <6 months
	APPSwe/PSEN1dE9 (Jankowsky et al. 2004)	Aβ deposits can be found by 6 months Abundant plaques in the hippocampus and cortex by 9 months Progressive increase in plaques up to 12 months. NFTs are absent Neuronal loss observed, specially in areas adjacent to plaques	Significant gliosis by 6 months, especially in areas around plaques	Impairment in the Morris water maze at 12 months
	5xFAD (Oakley et al. 2006)	Amyloid deposition starts at 1.5 months and is particularly high in subiculum and deep cortical layers. Aβ_42_ accumulates intraneuronally in an aggregated form within the soma and neurites starting at 1.5 months NFTs are absent Neuronal loss observed in cortical layer 5 and subiculum	Gliosis starts since 2 months of age and is proportional to Aβ_42_ levels and amyloid deposition. The number of activated astrocytes and microglia increases with age paralleling the age-related rise in amyloid burden	Impaired spatial memory as measured by the Y-maze test observed at 4–5 months
Aβ and tau	3xTg (Oddo et al. 2003)	Extracellular Aβ deposits observed at 6 months in the frontal cortex By 12 months, extensive tau pathology is present in CA1 neurons, and later in the cortex	Increased astrocyte and microglial density observed at 7 months of age	Cognitive impairment develops from 4 months of age and correlates with the accumulation of intraneuronal Aβ in the hippocampus and amygdala, although plaques and tangles are not apparent at this age
	APPSweDI/NOS2^−/−^ (Colton et al. 2008)	Increased Aβ_42/40_ ratio, as well as hyperphosphorylated and aggregated tau They exhibit considerable neuronal loss (30–40%) at the age of 12 months	Increased microglial density in brain areas associated with plaques and astrogliosis	Severe memory impairment

Studies carried out on APPPS1 mice show that their glial response is similar to that observed in humans. Activated microglia tend to cluster around Aβ plaques (Hefendehl et al. 2011), a phenomenon widely described in human AD (McGeer et al. 1987; Zotova et al. 2011, 2013; Serrano-Pozo et al. 2013) and also observed in other mouse models of AD-like pathology (Frautschy et al. 1998; Stalder et al. 1999). Of note, there may conceivably be significant differences between the animal models and human AD in the biochemical characteristics of plaques which influence the microglial phenotype; however, this seems to be a relatively under-explored area.

Curiously, although a locally increased microglial density was observed in the areas surrounding plaques (Hefendehl et al. 2011); microglial phagocytic activity and process motility were found to be impaired in the APPPS1 mice in association with amyloid plaques (Krabbe et al. 2013).

These results corroborate the previous findings on another mouse strain, the APPswe/PSEN1dE9 made by combining the human transgenes *APP* with the Swedish mutation and *PSEN1* with a deletion of the exon 9. In these mice, microglia clustering around amyloid deposits are dysfunctional and incapable of clearing amyloid (Hickman et al. 2008), and thus, the authors propose that the reduction in Aβ clearance may be due to changes in gene regulation. They also observed a two- to fivefold decrease in expression of microglial proteins known to bind Aβ and to contribute to its clearance, such as SRA (scavenger receptor A), CD36, and RAGE (receptor for advanced-glycation endproducts) (Hickman et al. 2008); whereas expression of the pro-inflammatory cytokines IL1β and TNFα was increased 2.5-fold. Therefore, this discrepancy between microglial function and inflammatory molecules secreted reasserts the dual character of microglia in AD. Indeed, on one hand, microglia appear to lose motility and sensitivity towards amyloid, which restrains them from performing their neuroprotective surveillance and clearance function; and on the other hand, microglia develop a pro-inflammatory profile that eventually results in neurotoxicity.

Studies using positron emission tomography (PET) imaging to trace amyloid and microglial activation have demonstrated that, like APPPS1 mice, AD patients have a significant increase in both amyloid and microglial signals, with the microglial signal particularly elevated in the vicinity of the amyloid signal (Edison et al. 2008). It is noteworthy that the authors described an inverse relationship between the microglial signal and cognitive performance, as measured by the mini-mental state examination (MMSE), which was not observed with the amyloid signal (Edison et al. 2008). Given the fact that amyloid deposition in AD patients commonly precedes the appearance of symptoms by decades (Okello et al. 2009; Serrano-Pozo et al. 2011a), this leads to the hypothesis that amyloid is likely not the direct cause of cognitive impairment in AD. In transgenic mice, the relationship between Aβ load and cognitive performance seems more straightforward (Bruce-Keller et al. 2011). A study performed in the PS1_M146L_+APP_K670N,M671L_ mice—human transgenes with the *APP* Swedish and the *PSEN1* M146L mutations, reported an association between Aβ_1–42_ load and memory performance using the water maze and radial arm tests (Gordon et al. 2001). Regarding the relationship between microglia and tau pathology in human brain, dystrophic microglia were found associated not only with amyloid deposits, but also with tau pathology suggesting a direct association between tau and microglia in AD (Streit et al. 2009). No relationship was observed between amyloid load and cognitive impairment on PET imaging (Edison et al. 2008) or with microglia in a post-mortem study (Serrano-Pozo et al. 2011b). Conversely, a direct relationship was present between the neurofibrillary tau pathology and the severity of cognitive deficits (Riley et al. 2002), and with microglial activation (Serrano-Pozo et al. 2011b). Interestingly, in a post-mortem study of 299 brains provided by the MRC-Cognitive Function and Ageing Studies (CFAS), investigation of the microglial immunophenotype in the elderly population showed that overall, in participants without dementia, negative relationships with the different pathological features of AD were prevailing; whereas in participants with AD, microglia were strongly associated with neuritic plaques and NFTs (Minett et al. 2016). Therefore, the association between microglia and AD pathology appeared to change its pattern between participants without and with dementia. It was also noted in this study that tangles were the only pathological feature related to cognition (Minett et al. 2016).

In the context of mouse models of AD, there is difficulty in assessing the relationship between microglia and tau, as the amyloid-centered models such as APPPS1 mice do not develop fibrillary tau pathology (Table 3), which may be due to their limited lifespan. Of note, the transgenic mouse models do not usually survived beyond 1 year of age. Other animal models have been developed which focus on tau pathology, such as the htauP301S and the PS19, which both carry the human *MAPT* (microtubule-associated protein tau) gene with mutations that cause fronto-temporal dementia. There is evidence that these models exhibit increased microglial activation in the CNS, with the human (h)tauP301S mice presenting activated microglia clustered around tau-positive neurons (Bellucci et al. 2004). It was demonstrated in PS19 mice that microglial activation precedes tangle formation (Yoshiyama et al. 2007), consistent with the previous observations of the presence of dystrophic microglia preceding the appearance of tau pathology in AD patients (Streit et al. 2009). From these observations, it can be inferred that microglia in transgenic mice behave in many ways similarly to human microglia regarding their relationship with AD pathological features. However, it should be kept in mind that tau-centered models do not exhibit amyloid deposition, and thus, their accuracy to reflect the changes that take place in AD brains is limited.

Some models have tried to replicate both Aβ and tau pathology in the mouse brain (Table 3).

One of these models was produced by crossing the transgenic lines Tg2576 (human APP with the Swedish mutation) with the JNPL3 which expresses the human *MAPT* with the P301L mutation to produce the APPSwe-Tau mice. These mice have a similar plaque distribution to the Tg2576 mice, e.g., plaques starting to appear at 6/7 months to become more consistent by 9 months spreading through the cortex and hippocampus. Interestingly, the NFTs appear before the amyloid plaques as early as 3 months to be widely present at 6 months, and were more severe than in the parental line JNPL3 (Lewis et al. 2001). Reactive microglia were observed from 3 months of age and clustered around plaques at 9 months (Lewis et al. 2001). This model by exhibiting more severe tau pathology supported a synergistic relation between both proteins. However, it is interesting to note that the tau pathology and microglial activation were concomitant. The widely used 3xTg-AD mouse strain bears human transgenes for APP, PS1, and tau with pathogenic mutations that lead to the development of both amyloid plaques and NFTs. Increased F4/80 microglia/macrophages were detected in the entorhinal cortex from the age of 6 months (Janelsins et al. 2005), as well as increased Iba1-positive microglia at a later stage in the hippocampus (Caruso et al. 2013). Another interesting mouse AD model, initially designed to explore the role of nitric oxide (NO) in the AD brain, reproduces both Aβ and tau pathology, and is known as APPsweDI/NOS2^−/−^ (Colton et al. 2008). This mouse produces the pathogenic APP Swedish, Dutch, and Iowa mutations and is knock-out for the mouse *nitric oxide synthase* (*NOS*)*2* gene, which encodes the inducible (i)NOS protein, an enzyme strongly associated with neuroinflammation and oxidative stress. In addition to the accumulation of human APP, these mice have high levels of mouse tau hyperphosphorylation and aggregation, increased microglial activation, neuronal loss, and severe memory impairment. The fact that the APPsweDI/NOS2^−/−^ mice, as the result of human amyloid accumulation and lack of *NOS2,* produce mouse tau pathology without the need of a human tau transgene, reveals a link between inflammation and tau pathology that very closely resembles findings from research in humans (Colton et al. 2008). It is apparent that the role of microglia, both in mice and humans, is highly dependent on the profile of activation that they display. Indeed, whereas microglia in normal conditions fulfil a neuroprotective role, in AD, their function leans more towards a neurotoxic pro-inflammatory status. To address this, the inter-species similarities and differences in microglial inflammatory profile will be discussed in the next section.

## Microglial inflammatory profile in AD

To understand the different activation patterns that microglia can adopt, it is important to consider the shared origin of microglia and macrophages. In vivo lineage tracing studies performed in mice have shown that microglia, as well as primitive macrophages, originate in the yolk sac derived from myeloid progenitor cells, and start differentiating as a distinct cell population early in embryonic development (Ginhoux et al. 2010).

Unlike macrophages, whose precursor cells, the monocytes, are constantly produced in the bone marrow, evidence indicates that microglia are mostly maintained throughout life (Ginhoux et al. 2010). Despite this essential difference, microglia and macrophages share many characteristics regarding gene expression, phenotype, and function. For example, microglia express many markers that are typically associated with macrophages, such as CD11b (Roy et al. 2006), CD14 (Cosenza et al. 2002), CSF1R (colony-stimulating factor 1 receptor) (Mizuno et al. 2011), CD68, HLA-DR, MSR-A, and FcγRI (Minett et al. 2016).

Regarding macrophage activation, traditionally, two distinct patterns have been described, commonly known as M1/classical or M2/alternative profile, with each one characterised by distinctive protein expression. This classification of macrophage activation has its origin in the nomenclature of T helper cells, in which distinct subsets (termed Th1 and Th2) can be recognised depending on the cytokines which they express after activation (Martinez and Gordon 2014). Further observations suggested that adopting a similar nomenclature for macrophages would be useful to understand their role in different conditions. Indeed, macrophages can react in several ways to diverse stimuli, and such responses can be considered as pro-inflammatory (M1) or anti-inflammatory (M2) based on cytokine expression.

M1 activation, also known as “classical activation”, is induced by IFN-γ and characterised by the production of pro-inflammatory cytokines, such as IL1β, IL6, MCP-1, and TNFα. On the other hand, M2 activation, or “alternative activation”, is defined by the release of anti-inflammatory cytokines such as IL4 and IL13 and increased expression of the inflammatory proteins AG1 (arginase 1), MRC1 (mannose receptor C, type 1), CHI3L1, and CH3L2 (chitinase 3-like 1 and 2) (Colton et al. 2006). The M2 profile is normally associated with tissue repair and reconstruction of the extracellular matrix (Bouhlel et al. 2007). A third profile of activation, generally considered a subtype of M2 activation, has been suggested and defined as “acquired deactivation”. This phenotype is mainly found in the presence of apoptotic cells and is characterised by the expression of IL10 and TGFβ, inhibition of the production of pro-inflammatory cytokines, and increased expression of scavenger receptors (Saijo and Glass 2011).

However, recent studies have found that, although this classification is still useful to some extent, in many situations, macrophage responses cannot simply be classified into M1 or M2. Studies on the macrophage transcriptome have pointed out that it is more accurate to consider macrophage activation as a spectrum, in which different transcriptional programmes operate in response to changes in the cellular environment (Xue et al. 2014). This is consistent with the previous observations that macrophages can express a wide range of receptors and inflammatory molecules (Mosser and Edwards 2008). This macrophage plasticity was suggested to also apply to microglia, in which expression profile studies show that in most pathological conditions, there is not a clear expression towards an M1 or M2 profile, but rather a mixed phenotype.

Indeed, Colton et al. explored the microglial profile in wild-type and AD transgenic mice ranging from day 3 to 12 months old. They did not observe the exclusive expression of either M1 or M2 markers whatever the age or strain of the animal. Indeed, the transgenic Tg2576 mice exhibited increased expression of TNFα, but also increased AG1 and MRC1. These results were consistent with the expression profile that they detected in the brains of AD patients, which showed increased expression of TNFα, AG1, CHI3L1, and CHI3L2 (Colton et al. 2006). Their findings demonstrated that microglial activation in AD cannot be regarded as exclusively M1 or M2, but that instead, both pro- and anti-inflammatory signals coexist in the same brain.

It was proposed that the microglial activation pattern in AD could be regarded as a mosaic (Colton et al. 2006), in which some microglia display an M1 phenotype and others an M2 phenotype, dependent on the microenvironment surrounding each particular cell. In a model of human microglial cells cultured and treated with Aβ, microglia actively performing phagocytosis had a lower expression of M1 markers, while M2 markers were increased (Hjorth et al. 2013). These results are relevant in the light of the findings discussed in the previous section regarding microglial motility and phagocytic activity, that microglial motility in AD transgenic mice is decreased and this impairment is spatially correlated with the presence of amyloid plaques (Krabbe et al. 2013). Therefore, M1 microglia might be less efficient in removing Aβ, indirectly contributing to Aβ accumulation (Hjorth et al. 2013). Thus, it is possible to hypothesize that microglia surrounding plaques have impaired motility and display an M1 phenotype, whereas microglia far from plaques develop an M2 phenotype. However, it is important to note that this in vitro study was performed on a human microglia cell line (Hjorth et al. 2013), and thus, the ex vivo cells might be more closely related to a macrophage than a microglial phenotype (Butovsky et al. 2014).

Nevertheless, following this idea of microglial activation as a spectrum rather a binary classification, it is acknowledged that to measure inflammatory cytokines is not sufficient to provide information about the activation pattern and the role performed by microglia in different conditions. Instead, this should be performed in association with the characterisation of the expression of proteins related to specific functions of microglia known as immunophenotyping.

Apart from microglial cells, there is debate about the role that peripheral monocytes/macrophages infiltrating from the peripheral blood might play in AD. Whether these cells may be relevant for the immune response in the brain and if their participation may be beneficial or detrimental in the disease pathogenesis is still unclear (Simard et al. 2006; Gate et al. 2010) and needs to be further studied in both humans and mice.

To summarise this section, there is a consensus regarding the concept that microglia, like the macrophage, can adopt several inflammatory phenotypes, resulting from genetic and environmental stimuli. However, our knowledge and understanding of these profiles in human conditions still remain to be fully characterised (Table 4).

**Table 4:** Comparison of ageing-related differences between mice and humans

Organism	Human	Mouse
Life span	Life expectancy is at least 81 years in several countries, with an increase by 2–3 months every year without any indication of a slow-down (Global Burden of Disease Study 2015)	Life span between 2–3 years depending on strain (Yuan et al. 2011)
Brain volume loss	Significant brain volume loss associated both with normal and pathological ageing, although it is exacerbated in pathological conditions (Schuff et al. 2012)	Total brain size increases with age in wild-type mice (Maheswaran et al. 2009)
Microglial morphology	Primed microglia with greater immunoreactivity Dystrophic microglia with fragmented processes (Streit et al. 2009)	Thickening and a deramification of microglial processes, with hypertrophy of the cell body (Hart et al. 2012)
Inflammatory profile	Increased expression of IL-1, IL6, TNF-α, TGFβ, chemokines, complement proteins, and TLRs (Lopez-Gonzalez et al. 2015)	Increased expression of cytokines and inflammatory mediators, in a pattern very similar to the human (Lopez-Gonzalez et al. 2015)

## Microglia and cognition

Clinically, AD symptoms include deficits in cognitive functions such as episodic, visuospatial, and semantic memory, with language, reasoning, and decision-making also affected in patients. In the human brain, these functions heavily rely on interactions between the hippocampus and the neocortex (Mummery et al. 2000). Compared to the murine neocortex, the human neocortex is considerably larger in size and more complex in terms of the cell number and their connections (Lui et al. 2011). For this reason, the complexity and range of human cognitive functions are difficult to replicate and evaluate in a small rodent. Nevertheless, using methods such as passive avoidance and maze-based tasks can shed some light as to whether some of the cognitive symptoms of the disease are mimicked in the animal model.

Different studies have evaluated the relationship between inflammation and cognition in both normal and pathological conditions. Many pro-inflammatory cytokines, such as IL1β, IL6, and type I and type II interferons, disturb long-term potentiation (LTP), a mechanism involved in learning and memory. Interestingly, LTP was observed to be diminished in aged mice (Griffin et al. 2006), likely as the result of the increased pro-inflammatory cytokines characteristic of ageing (Lopez-Gonzalez et al. 2015). Studies have also evaluated the relationship between microglia and cognition in pathological conditions. In transgenic AD models, the majority of the studies have identified microglial activation as a factor that contributes to neurodegeneration and accelerates cognitive deterioration. In APP SwePSEN1dE9 mice, poor performance in the Morris water maze was associated with increased microglial activation and IL1β brain levels (Gallagher et al. 2013). Overall, high levels of pro-inflammatory cytokines have been related to cognitive impairment in various studies in both mice and humans (Munoz et al. 2007; Swardfager et al. 2010). Conversely, pharmacological inhibition of microglial activation, for example, with minocycline (Biscaro et al. 2012), blocking IL1β signalling (Kitazawa et al. 2011), or inhibition of TNFα expression (Chavant et al. 2010), has been reported to reduce inflammation and improve cognitive performance in AD transgenic mice.

On the other hand, some studies have highlighted a beneficial role of activated microglia for cognitive function based on their inflammatory profile. In APPswe/PS1dE9, AD mouse model with a mutation in the toll-like receptor (*TLR*)-*4* gene (causing a loss of function of this protein) decreased microglial activation was observed, with increased Aβ deposition and aggravation of the cognitive deficits (Song et al. 2011). These findings suggested that activated microglia might be neuroprotective in some circumstances, at least through TLR4.

Similarly, it was observed that in APPSwePSEN1dE9 mice, the induction of peroxisome proliferator-activated receptor (PPAR)γ that activates microglia by suppressing inflammatory gene expression and enhancing phagocytosis resulted in a reduction of Aβ in the hippocampus and improvement of cognitive functions (Yamanaka et al. 2012). Interestingly, treatment of APP23 mice with the anti-inflammatory cytokines IL4 and IL13 led to an amelioration of cognitive deficits (Kawahara et al. 2012). This indicated that in mice, microglial activation, when it leans toward an M2 phenotype, seems to promote Aβ clearance resulting in a beneficial effect for cognitive function.

In humans, we previously mentioned the PET imaging study showing an association between the microglial PET signal and the cognitive decline in living AD patients (Edison et al. 2008). In the recent post-mortem MRC-CFAS study mentioned in the pathology section, the authors also observed that with ageing, microglia lose the Iba1 protein associated with motility, likely necessary to support neurons, and this was associated with the development of dementia. Conversely, other microglial proteins (CD68, Macrophage Scavenger Receptor (MSR)-A), the role of which is clearance of damaged cellular material, were associated with the development of dementia and poor cognition (Minett et al. 2016). This study highlighted that different microglial functions can coexist within the same brain. The mouse and human studies clearly support the concept that microglial activation affects cognition, in keeping with the genetic findings indicating risk for AD associated with inflammation-related genes. Discrepancies in the inflammatory profile detected in the animal studies, may be due to the differences in the mutations used to develop the transgenic models (Morgan et al. 2005). In humans, we still do not know the precise inflammatory profiles present at the onset and during progression of the disease. Such profiles might result from the sum of (1) pre-existing variation in genes related to inflammation, (2) microglia primed by diverse systemic infections experienced during life, and (3) combinations of pro and anti-inflammatory molecules produced by microglia responding to different aspects of AD pathology. PET imaging is a very useful tool to allow studies of microglia at serial time-points in living patients, but the microglial PET compounds currently available do not yet allow the characterisation of the phenotype of the ongoing inflammation.

The hypothesis that microglial activation could be beneficial in AD due to its effect on amyloid removal by phagocytosis is relevant to strategies that involve active or passive immunisation directed against Aβ or tau. Immunisation studies in AD mice have provided interesting and promising results, with many of them reporting beneficial effects from the treatment. Anti-Aβ immunisation was successful in decreasing amyloid plaque burden and this was facilitated by activated phagocytic microglia (Schenk et al. 1999; Wilcock et al. 2003, 2004a). In mouse models, Aβ clearance had multiple effects, including recovery of synapse and neurite morphology (Lombardo et al. 2003; Buttini et al. 2005; Brendza et al. 2005; Spires-Jones et al. 2009) and improved cognitive performance (Morgan et al. 2000; Janus et al. 2000; Wilcock et al. 2004b; Rasool et al. 2013). Immunotherapy against tau in tau-based model of rodents (Chai et al. 2011) induced decreased NFT burden and restored cognitive function (Asuni et al. 2007; Boimel et al. 2010; Boutajangout et al. 2010; Chai et al. 2011). Interestingly, one study did observe increased microglial activation (Boimel et al. 2010); whereas two other studies did not see changes in microgliosis (Boutajangout et al. 2010; Chai et al. 2011), possibly due to the different models and time-points examined (Chai et al. 2011).

However, clinical trials involving Aβ immunotherapy in humans have not been as successful as studies in mice. The first clinical trial was an active protocol with the use of the full-length preaggregated synthetic Aβ_42_ (Bayer et al. 2005) named AN1792. A long-term clinical-neuropathological follow-up of the patients showed continued cognitive decline despite clearance in amyloid plaques in some patients (Holmes et al. 2008). Other clinical trials of passive immunisation using humanized anti-amyloid antibodies bapineuzumab (Salloway et al. 2014) or solanezumab (Doody et al. 2014) have demonstrated no clear effect in improving cognitive function or slowing rate of decline in participants with established AD. However, analysis of the solanezumab data in people with mild AD showed a trend toward improved cognition moving the field of immunotherapy in AD from established AD towards the prodromal or mild AD with the aim to halt the cognitive decline as early as possible. A recent trial with the aducanumab antibody seems to confirm the slowing of the cognitive decline in patients with mild AD (Sevigny et al. 2016).

Tau-directed immunotherapy has been initiated and compounds for PET imaging of tau are currently being developed (Sigurdsson 2016). The first clinical trial of a phosphorylated tau immunogen has been completed regarding the safety, tolerability, and immunogenicity of the compound, and others trials are already underway (Pedersen and Sigurdsson 2015), but the findings have not been published yet.

A remarkable observation from the human Aβ immunisation studies, particularly with AN1792, is the fact that post-mortem analysis revealed that immunisation was effective in terms of reducing amyloid load via enhanced Aβ phagocytosis by microglia (Nicoll et al. 2006; Zotova et al. 2011), with a possible contribution from other mechanisms, as observed in the animal studies. Notwithstanding, this was not correlated with improvement of cognitive performance (Holmes et al. 2008), corroborating the previous studies that pointed out the lack of association between amyloid pathology and severity of dementia (Davis et al. 1999; CFAS 2001; Engler et al. 2006; Minett et al. 2016). In this regard, it is pertinent to highlight the hypothesis stated by Serrano-Pozo et al. (2011b) that a certain threshold of amyloid accumulation may be required to trigger a microglial response and initiate a pathogenic cascade, likely involving tau, beyond which neurotoxicity cannot be reversed simply by removing the amyloid. This reminds us that genetic studies, which highlight the features of disease that are causes rather than effect, have emphasized a role for inflammation in the disease pathogenesis.

Overall, mice can form the basis of valuable studies of behaviour in selected aspects of microglial responses and pre-specified features of AD pathology. However, limiting factors include the higher complexity and structural differences of human neural networks compared with mice, the difficulty in modelling the full spectrum of AD pathology, the less severe neurodegeneration, and better capacity for recovery in the animal models.

## Conclusion

A number of similarities exist between murine and human microglial cells supporting the importance of the animal studies. These have been and are extremely useful to identify potential roles or pathways involving microglia during development, and in healthy and pathological conditions, with some of them already validated in the human brain. However, the differences between microglia of a laboratory animal reared in pathogen-free conditions and those in a human patient complicate the translation of the research to the human brain. There are fundamental differences between mice and humans, and thus, more exploration in the human brain is needed to assess the relevance of the models and guide their further development. Meanwhile, the experimental findings should be interpreted paying close attention to the limitations of the models utilised.
